# Randomly distributed optical fibers in translucent mortar for privacy-preserving light transmission and digital image reconstruction

**DOI:** 10.1038/s41598-025-32224-2

**Published:** 2025-12-24

**Authors:** Karina Hwang Arcolezi, Vivien Marion, Bora Ung, Claudiane Ouellet-Plamondon

**Affiliations:** 1 Department of Construction Engineering, École de Technologie Supérieure, Université du Québec, Québec City, Canada; 2 Department of Electrical Engineering, École de Technologie Supérieure, Université du Québec, Québec City, Canada; 3 Université de Bordeaux, INP Enseirb-matmeca, Talence, France

**Keywords:** Light-transmitting materials, Image reconstruction, Visual privacy, Physical-digital interface, Engineering, Materials science, Mathematics and computing, Optics and photonics

## Abstract

Light-transmitting building materials often compromise visual privacy due to coherent light transmission. This study presents a novel composite utilizing randomly distributed optical fibers coupled with a computational image reconstruction system. A limestone-calcined clay cement (LC³) inspired matrix was designed for sustainability and material performance. An algorithmic approach assigned a random traceable fiber distribution via a bijective input-output mapping. The random fiber configuration achieves effective light diffusion, preserving physical privacy. However, using digital imaging and homography-based calibration, the network was computationally reconstructed to reverse the diffusion, recovering hidden visual information accurately. This demonstrates a dual functionality: architectural privacy combined with selective digital transparency. Geometric robustness tests confirmed a stable operational envelope (estimated error of 2.9%) across viewing distances of 30–110 cm and camera rotation angles up to ± 35º (pitch and yaw), establishing these fiber-instrumented cementitious composites as hybrid physical-digital materials for smart infrastructure applications.

**Supplementary Information:**

The online version contains supplementary material available at 10.1038/s41598-025-32224-2.

## Introduction

 The incorporation of light-transmitting elements in construction materials has been studied as a strategy to improve daylight utilization, reduce dependence on artificial lighting, and enhance the aesthetic performance of buildings^1–7^. Various approaches have been explored to achieve this goal, including the use of glass blocks, translucent polymers, and cementitious composites with embedded inclusions designed to guide or diffuse light^8–10^. Together, the development of the cementitious matrix itself is moving toward low-carbon alternatives, such as Limestone Calcined Clay Cement (LC^3^), which offers a sustainable base for high-performance architectural materials^11^. These solutions often seek to balance structural performance with visual effects, offering both functional and architectural benefits.

Among the different methods, the incorporation of optical fibers into cementitious matrices has attracted particular attention^12,13^. Optical fibers transmit light efficiently across relatively long distances while maintaining their flexibility and compatibility with casting processes^6,14,15^. When embedded in mortars or concretes, they enable external light to penetrate through solid, opaque elements, creating composites that combine mechanical strength with the ability to channel illumination into interior spaces^7,16–19^.

Most studies in this field have focused on configurations where fibers are placed in straight and parallel arrangements^15,17–25^. This design ensures efficient light transfer and, in many cases, allows the perception of shapes or silhouettes from the opposite side of the material^2,26^. While this property produces distinctive visual outcomes, it also introduces a limitation. In applications where privacy is required, the transmission of recognizable contours may restrict the use of these composites.

In recent years, the development of light-transmitting concretes has also intersected with broader research on smart and responsive materials, where optical components are used not only for illumination but also for information capture and visualization^27–29^. Such materials open perspectives for real-time monitoring, imaging, and augmented-reality applications in construction, enabling interactive or perceptive building elements.

In this work, we propose a different approach. Instead of a parallel arrangement, fibers are introduced in a random distribution that modifies the way light propagates through the material. This strategy produces a composite that is partially translucent while reducing the possibility of visual recognition of shapes and objects behind it. This design is conceived as a functional material for architectural privacy, but also as a physical platform for optical data transmission and digital imaging experiments. Beyond the physical development of the composite, we also explore a digital perspective. By mapping the position of each fiber, it becomes possible to reconstruct them computationally into ordered virtual paths. This process, which we define as a type of digital reverse engineering, makes it feasible to reconstruct visual information hidden in the physical specimen. The main objectives of this study are therefore twofold:

i) To develop a mortar-based light-transmitting composite with randomly distributed optical fibers, capable of combining light transmission with privacy;
ii) To establish a digital methodology for the geometric reverse engineering of the randomized fiber network, demonstrating that the diffused light can be computationally reconstructed to recover hidden visual information.

By combining physical material design and digital reconstruction, this study proposes a new paradigm where the composite functions as an active physical-digital interface, transforming a passive architectural component into a functional optical processor. This dual functionality positions the composite both as a privacy-preserving material and as a platform for selective visual information retrieval, highlighting its potential as an active physical-digital component in architectural applications. This approach aims to expand the potential of translucent composites in architecture, where both light transmission and privacy represent key features.

### Conceptual design of the semi-translucent composite with privacy

**Fig. 1:**
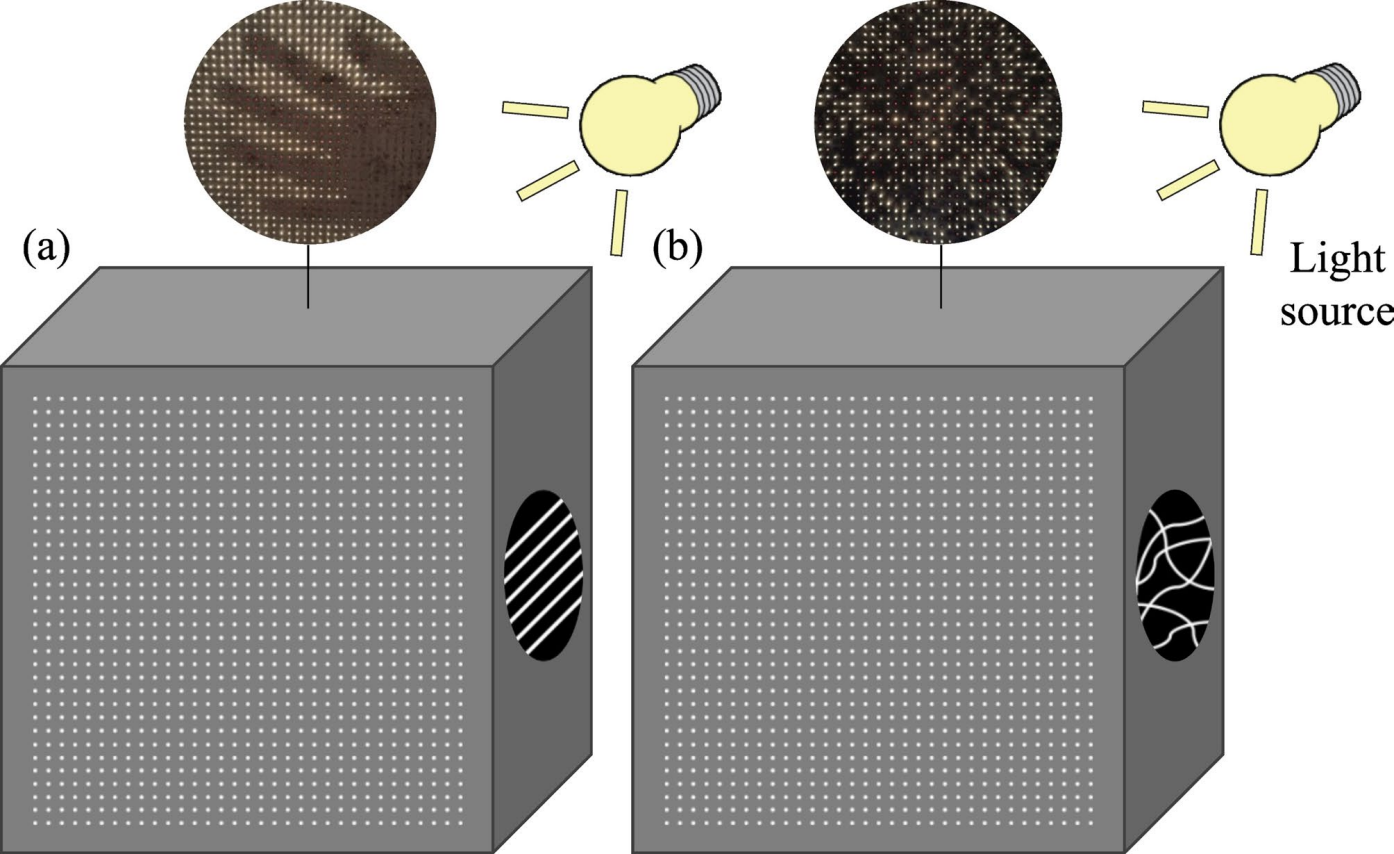
Comparison between (**a**) a conventional light-transmitting composite with parallel fibers, and (**b**) the proposed random fiber distribution, which diffuses light and maintains privacy.

In conventional light-transmitting concretes, the optical performance is influenced primarily by the geometric configuration of the fibers, which are commonly embedded in straight and parallel distribution (Fig. 1)^2,30–32^. This arrangement creates direct optical channels between the two faces of the element, allowing light to pass with limited deviation from its original direction. Several studies report that such parallel configurations can preserve a degree of spatial correspondence between the illuminated and receiving faces, making it possible in many cases to perceive general contours or shapes through the panel^2,26^. Although the extent of this visual coherence depends on several parameters, such as fiber density, diameter, spacing, and lighting conditions, straight-line arrangements tend to maintain a recognizable structure in the transmitted light^2,33^. From an architectural standpoint, this can be desirable to convey visual information or enhance transparency, but it may also limit the use of these systems in contexts where privacy is required.

To address these limitations, the design proposed in this study diverges from the conventional approach by introducing fibers with no predefined alignment, crossing the specimen along irregular paths. These trajectories interrupt direct optical channels and disperse the transmitted light, reducing coherence and preventing the formation of discernible shapes. The resulting composite combines two functions, allowing the passage of light while suppressing visual perception, which creates a pathway toward privacy-oriented translucent architectural elements.

Previous studies have also explored randomly distributed fibers, often referring to a non-uniform planar arrangement^19^, but with parallel, linear trajectories through the specimen, and almost exclusively to characterize their physical properties, such as fiber orientation^15^, intersection density, or network connectivity for understanding material formation or mechanical performance^28^. These efforts treated randomness as a structural descriptor rather than as an optical mechanism.

In addition to studies focused on optical, mechanical, or structural properties, other research has investigated the reconstruction and analysis of fiber network topology using image-based approaches. Tang et al.^34^ and D’Amore et al.^35^ demonstrated that imaging and reconstruction techniques can be used to characterize and modify complex fibrous networks through descriptors such as connectivity, orientation, and branching. Although these works are not related to optical transmission, they highlight the broader applicability of image analysis for extracting and reorganizing information from disordered fiber structures. Within this context, similarity metrics commonly used in image analysis, such as Normalized Cross-Correlation (NCC), provide a quantitative means to evaluate spatial correspondence between images or reconstructed patterns^36–39^.

Other approaches, such as Li et al.^40,41^. and Zheng et al.^42^. have developed algorithms for 3D reconstruction of granular or fibrous networks and dense image segmentation using vision-based models. Although these methods focus on granular media or biological tissues rather than light transmission, they highlight the utility of computational reconstruction and topological analysis to recover structural information from complex, irregular networks. Such concepts support the broader relevance of digital mapping and reconstruction strategies for fiber-based materials.

Accordingly, while these studies incorporate a form of randomness, the random three-dimensional trajectories as a means to disrupt optical coherence or enable digital reconstruction intentionally are not employed, which constitutes the main conceptual contribution of the present work. In contrast, the proposed design treats the random network not as a property to be analyzed, but as a physical encryption layer: the random paths intentionally diffuse light in the physical domain while remaining traceable for digital reconstruction.

Figure 1 compares both configurations. In conventional design (Fig. 1a), parallel fibers transmit light directly, preserving shapes. In the proposed configuration (Fig. 1b), randomly oriented trajectories scatter the transmitted light, preventing image formation while maintaining brightness. The irregular fiber paths also generate a complex internal pattern that can later be captured, analyzed, and reconstructed digitally.

The random distribution was not produced arbitrarily but generated through a computational algorithm that assigns a unique correspondence between each entry and exit point of the fibers. This approach ensures that randomness is fully traceable and reproducible, allowing the physical arrangement to be digitally reconstructed afterward. Each fiber, therefore, carries both an optical and an informational role, acting as an individual data channel within a randomized network.

**Fig. 2:**
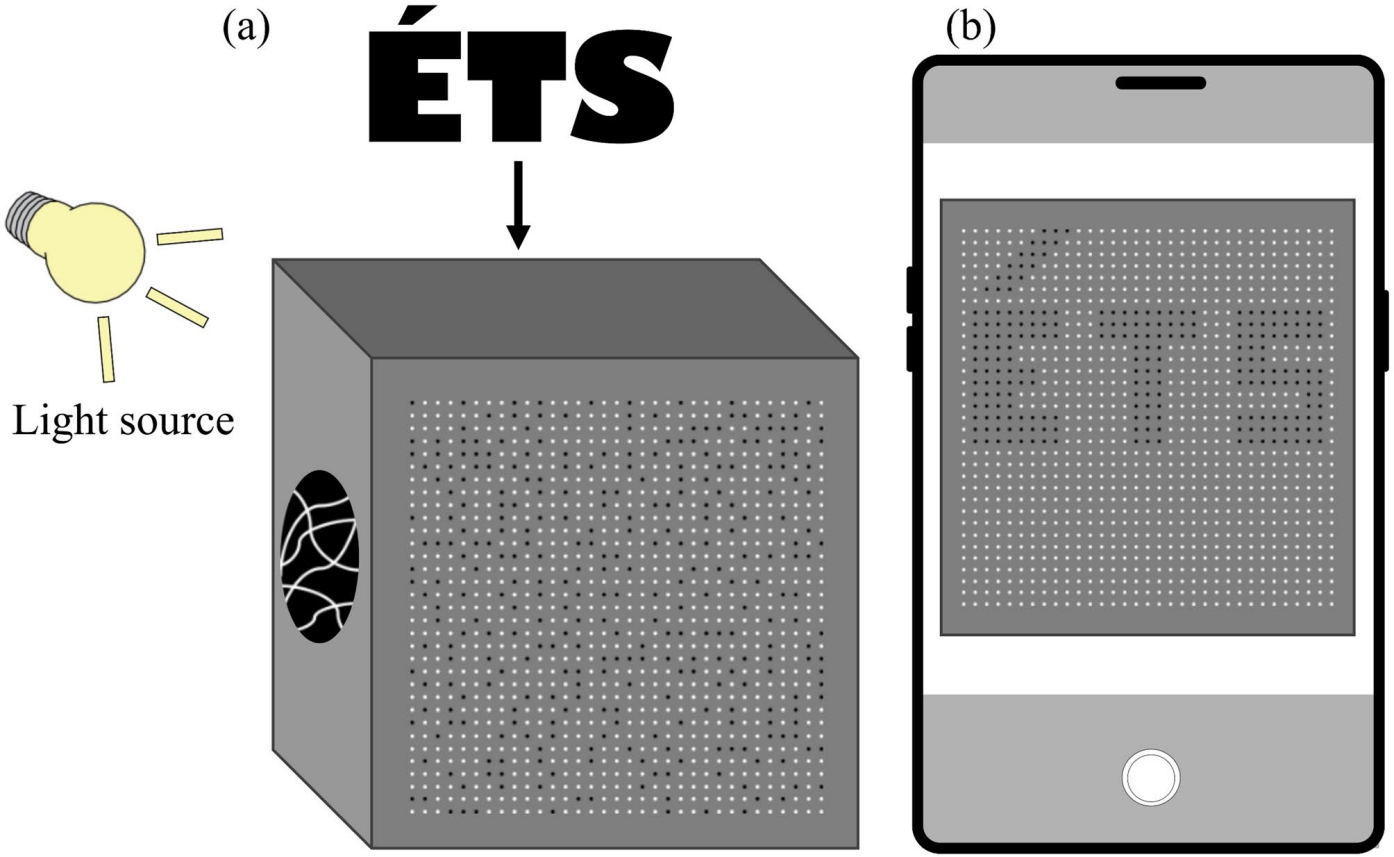
Digital reconstruction of fiber paths enables recognition of hidden letters “ÉTS” that remain indistinguishable in the physical specimen.

This property enables a second level of analysis. The internal network of fibers can be mapped not only as spatial geometry but also as a framework for light transfer. Once recorded, the random paths may be computationally reconstructed into virtual straight lines, creating a digital version of the specimen with ordered transmission channels. In this virtual configuration, light emerging from random exit points is reassigned to coherent paths, which makes it possible to reconstruct images hidden by the physical material. Figure 2 illustrates this principle. A background object, represented by the letters “ÉTS”, remains indiscernible in the physical sample, yet becomes visible when the recorded fiber network is reconstructed digitally.

In summary, the proposed design replaces parallel fiber arrangements with traceable random trajectories, enabling light transmission while suppressing visual recognition at the material level. At the same time, the preserved fiber mapping allows digital reconstruction, defining the composite as a physical-digital optical interface for privacy-oriented architectural applications.

## Materials and methods

### Fabrication of the semi-translucent composite with privacy

The cementitious matrix material used was a limestone calcined clay cement (LC^3^) inspired mortar^11^ (Table 1). The selection of LC^3^ was based on a dual requirement of sustainability and material performance. From a sustainability perspective, this system enables a clinker replacement exceeding 40%^11,43^, reducing the use of Portland cement, which is a contributor to global CO_2_ emissions from the construction sector^44^. In addition, the chemistry of LC^3^ provides better control over the rheology and microstructure of the fresh mortar. The reaction between the alumina in the calcined clay and the calcium carbonate in the limestone leads to the precipitation of additional hydrates, notably carboaluminates and ettringite^43,45^. These reactions contribute to the final mechanical performance and to microstructural refinement, improving the balance between viscosity and flowability for effective infiltration of the dense fiber network.

Ordinary Portland cement (Type GU, CSA A3000^46^) was used as the primary binder. Supplementary cementitious materials included calcined clay (CC) and limestone filler (LF), following mix design principles established for LC^3^ systems^11,43,47,48^. Natural river sand (Sa), with a maximum particle size of 2.5 mm, was used as a fine aggregate. A polycarboxylate-based superplasticizer (SP) was incorporated to achieve the required workability. Mortar mixing was performed per ASTM C305^49^, and specimens were cast and cured following ASTM C109-21^50^ standard practices. The mix design aimed to produce a matrix compatible with optical fiber embedding, ensuring sufficient viscosity to prevent fiber displacement during casting while maintaining adequate flowability to penetrate the dense and entangled fiber network. Preliminary tests were performed to adjust the flow value for controlled casting without segregation, as proposed by Jin et al.^11^. Specimens were demolded after 24 h and cured in a humidity chamber (23 ± 2 °C, RH > 95%) for 28 days prior to mechanical and optical testing.

**Table 1:** Composition and properties of the partially translucent material.

OPC	CC	LF	Sa	Water	SP	Flow*	Density	Compressive strength**
kg/m³						%	kg/m³	MPa
390	162.5	97.5	1310	255	15	152	2210	49.9 ± 0.2

The light-transmitting elements were polymethyl methacrylate (PMMA) optical fibers (Eska™, Mitsubishi Chemical). Each fiber had a core diameter of 1 mm, a core refractive index of 1.49, and a numerical aperture of 0.5.

### Random fiber distribution and algorithmic traceability

To achieve a traceable random distribution of the optical fibers, a computational algorithm was implemented. The mold consisted of two parallel acrylic plates, face A (entry) and face B (exit), each containing a 33 × 33 grid of potential fiber positions, spaced at a centre-to-centre distance of 0.5 cm. Each position was indexed according to a coordinate system where the first two digits represented the row and the last two digits the column of a matrix. For each fiber, the algorithm sequentially selected coordinates on faces A (A0101 to A3333) and assigned a random, non-repeating exit coordinate on face B. This procedure utilized all 33² = 1089 entry points and ensured that the irregular path of each fiber remained fully traceable through a recorded input-output mapping matrix *M* (Eq. 1).

1 $$\:M=\left[\begin{array}{cc}{A}_{1}&\:{B}_{r\left(1\right)}\\\:{A}_{2}&\:{B}_{r\left(2\right)}\\\: \vdots &\: \vdots \\\:{A}_{n}&\:{B}_{r\left(n\right)}\end{array}\right], N=1089$$

Where *A*_*i*_ represents the coordinate index of the *i-th* fiber on face A, and *B*_*r(i)*_ denotes its randomly assigned exit coordinate on face B, determined by a permutation function *r*_*(i)*_ such that *r*_*(i)*_ is a bijective mapping of {1, …, n} onto itself. This bijective mapping defines a unique correspondence between entry and exit points, encoding both geometric randomness and complete traceability of the fiber network. The resulting dataset later serves as the geometric basis for the digital reconstruction and homography alignment^52,53^.

**Fig. 3:**
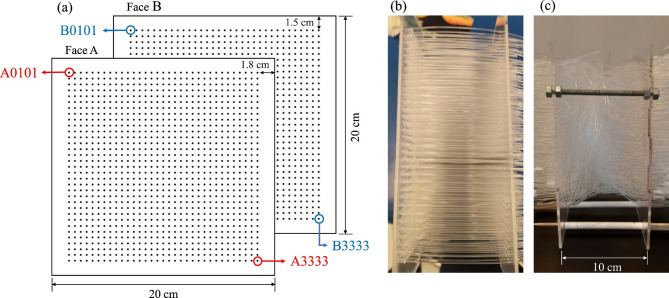
Representation of the fiber distribution system. (**a**) Schematic of the acrylic face A and B, (**b**) mold with fibers placed straight, and (**c**) mold with fibers placed randomly.

Following the dataset defined by the mapping matrix *M*, fibers were manually threaded through the mold and fixed before mortar pouring. This configuration was therefore both random in the geometric arrangement of fibers, and controlled through the algorithmic mapping that guaranteed complete traceability. A control specimen containing the same number of fibers aligned in straight, parallel paths was also fabricated for direct comparison of light diffusion and privacy performance (Fig. 3).

To preserve the integrity of the fiber network during casting, the fibers were mechanically constrained by the rigid acrylic mold plates. Since the fibers pass through fixed grid holes, the mapping matrix *M* is defined strictly by these fixed boundary coordinates. Therefore, regardless of minor internal displacements caused by mortar flow or hydration, the input-output correspondence remains consistent. To further secure this arrangement, the fibers were prepared with a significant excess length relative to the mold depth (Fig. 3c), providing sufficient margin to prevent tension forces from dislodging the fiber ends during casting. Finally, the rheology of the LC^3^ matrix was optimized to achieve close to a self-compacting consistency, allowing the mortar to infiltrate the entangled fiber network under gravity without the need for heavy vibration, thus minimizing the risk of fiber breakage or displacement^15^.

### Digital reconstruction and reverse engineering

#### Image acquisition and calibration

For the digital reconstruction, images of the specimen’s exit face (Face B) were acquired using a portable camera connected to a system running *MATLAB* (including the Computer Vision Toolbox).

**Fig. 4:**
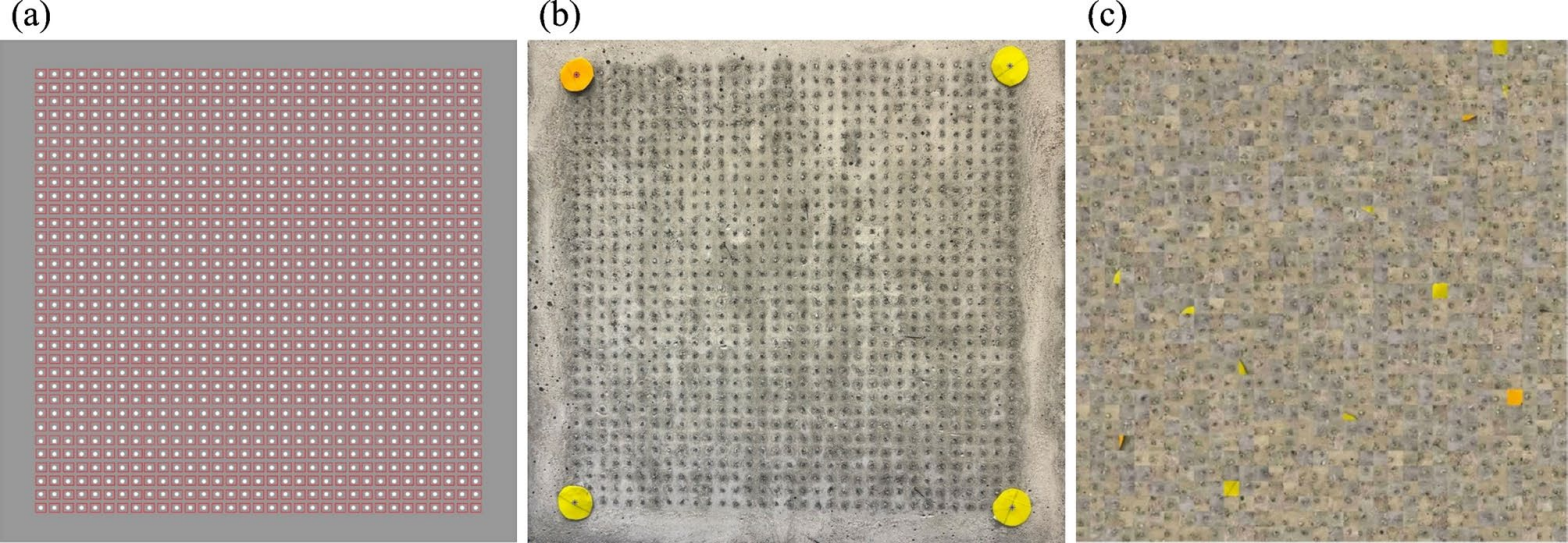
Digital acquisition and calibration of the fiber grid: (**a**) quadrangular areas assigned to each fiber; (**b**) coloured markers placed at the corners of the grid for calibration; (**c**) reconstructed view of the grid after homography transformation.

To calibrate the image and correct for perspective distortions, four coloured markers were positioned at the corners of the 33 × 33 grid. A preliminary colour study confirmed that yellow and orange offered the highest visibility and contrast under variable lighting conditions. The selection criteria involved maximizing a visibility metric, defined as the average sum of saturation and luminosity for each tested colour. To manage camera rotations exceeding 90º, one marker was assigned a distinct colour (orange), serving as an orientation reference to prevent misidentification (Fig. 4b).

#### Automated calibration via homography transformation

The geometric correspondence between the fiber exit grid (Face B) in the image space and the theoretical coordinate system was established through a Homography Transformation Matrix H, similar to that proposed by Wang^52^. This is important for correcting perspective distortion caused by camera position and angle. The homography matrix *H* relates a point x₁ = (x_1_, y_1_, 1)^T^ in the image (distorted) plane to its corresponding point x₂ = (x_2_, y_2_, 1)^T^ in the rectified (orthogonal) plane (Eq. 2).

2 $$\:H=\left(\begin{array}{ccc}{h}_{11}&\:{h}_{12}&\:{h}_{13}\\\:{h}_{21}&\:{h}_{22}&\:{h}_{23}\\\:{h}_{31}&\:{h}_{32}&\:{h}_{33}\end{array}\right)$$

Considering four pairs of points, denoted as (x_1_, y_1_) and (x_2_, y_2_) for images A and B, respectively, which define a quadrilateral in each image. The correspondence between these coordinates and the coefficients of the homography matrix *H* is then established as Eq. (3).

3 $$\:\left\{\begin{array}{c}{x}_{2}=\frac{{h}_{11}{x}_{1}+{h}_{12}{y}_{1}+{h}_{13}}{{h}_{31}{x}_{1}+{h}_{32}{y}_{1}+{h}_{33}}\\\:{y}_{2}=\frac{{h}_{21}{x}_{1}+{h}_{22}{y}_{1}+{h}_{23}}{{h}_{31}{x}_{1}+{h}_{32}{y}_{1}+{h}_{33}}\end{array}\right.$$

Assuming h_33_ = 1, this relationship can be expressed in linear form as AX = B, where $\:X={\left[{h}_{11}\:\:\:\:{h}_{12}\:\:\:\:{h}_{13}\:\:\:\:{h}_{21}\:\:\:\:{h}_{22}\:\:\:\:{h}_{23}\:\:\:\:{h}_{31}\:\:\:{h}_{32}\right]}^{T}$, and matrices A and B are constructed from the coordinate pairs of the corresponding points. The coefficients of H are solved by least-squares estimation, and the inverse transformation of $\:{H}^{-1}$ satisfies $\:H\cdot\:{H}^{-1}=I$. This homographic model establishes the spatial consistency necessary to ensure that the reconstructed fiber network accurately reflects the real geometry of the specimen, regardless of camera orientation.

Specifically, while the pre-recorded mapping matrix (M) establishes the logical inverse relationship between fiber coordinates, a computational algorithm is essential to bridge the gap between this theoretical map and the dynamic, real-world image capture. The algorithm serves three main functions: first, it performs image processing and segmentation to accurately identify the discrete light signature of each fiber on the physical exit surface. Second, it calculates a homography transformation matrix (H) to dynamically correct the captured image for real-world perspective and projection distortions (camera angle, distance). This step is necessary to ensure the physical light patches are precisely aligned with the theoretical grid defined in M. Only after these geometric and photometric corrections are applied can the system utilize M to reassign the image patches to their virtual, ordered coordinates, rendering hidden objects visible^53^.

#### Digital reconstruction algorithm

The core of the reconstruction relied on the rectified image of face B and the pre-recorded mapping matrix *M*. The reconstruction procedure involved two steps for each frame:

i) Patch assignment: after image rectification, a small quadrangular image area (patch) was digitally assigned around the exit point of each of the *N* = 1089 fibers (Fig. 4a). This patch represents the light signal transmitted through that specific fiber.
ii) Virtual realignment: the *N* image patches were then repositioned into a new, ordered virtual grid according to the inverse logic of the mapping matrix *M* (Fig. 4c). For every fiber i, the light patch recorded at the random physical output coordinate $\:({B}_{{x}_{i}^{{\prime\:}}},{B}_{{y}_{i}^{{\prime\:}}})$ was computationally reassigned to the coherent virtual output coordinate $\:({B}_{xi},{B}_{yi})$. This process digitally reverses the scattering effect, converting the physically diffused light pattern into a coherent visual image.

#### Normalized 2D cross-correlation

To quantify the similarity between predefined templates and the target images (reconstruction and privacy-preserving images), this study utilizes the normalized cross-correlation (NCC) function. This technique serves to confirm the fidelity of the reconstructed images by successfully matching known templates taken from a reference image (e.g., the letters ‘É’, ‘T’, ‘S’), and assessing the effectiveness of the privacy by observing the maximum correlation score, where a lower score indicates greater privacy protection.

NCC is a widely used measure for template matching in computer vision^36–39^. It quantifies the linear relationship between a template T and a patch of an image I (of the same size as T) by normalizing the cross-correlation result. This normalization makes the method robust to changes in image lighting and exposure. The NCC coefficient, $\:R(u,v)$, at a location $\:(u,v)$ in the image I, is computed as Eq. (4).

4 $$\:R\left(u,v\right)=\frac{{\sum\:}_{x,y}\left[I\left(x,y\right)-{\stackrel{-}{I}}_{u,v}\right]\cdot\:\left[T\left(x-u,y-v\right)-\stackrel{-}{T}\right]}{\sqrt{\sum\:_{x,y}\left[I\left(x,y\right)-{\stackrel{-}{I}}_{u,v}\right] ^{2} \cdot\:\sum\:_{x,y}\left[T\left(x-u,y-v\right)-\stackrel{-}{T}\right] ^{2} }}$$

Where $\:\stackrel{-}{T}$ is the mean intensity of the template, and $\:\stackrel{-}{I}(u,v)$ is the mean intensity of the image patch at position $\:(u,v)$. The resulting coefficient ranges from − 1 to 1, with 1 indicating perfect correlation. In this study, for each template, the maximum NCC value within the target image is recorded as the similarity score. High values indicate successful template reconstruction, while lower values in privacy-preserving images indicate effective obfuscation. By analysing the maximum correlation value, this quantitative approach allows an objective assessment of both reconstruction fidelity (higher values) and privacy effectiveness (lower values).

#### Real-time processing and geometric robustness tests

To assess the dynamic performance and the system’s robustness for practical applications, the entire processing pipeline was tested under two conditions:

i) Dynamic scene test, where the specimen was placed in front of a moving object (e.g., a hand opening and closing). The motion was captured as a video sequence, and the algorithm was applied frame-by-frame to demonstrate the temporal fidelity of the reconstructed image, verifying the system’s capacity to track rapid changes.
ii) Handheld acquisition test, where the reconstruction algorithm was implemented using a live acquisition system, where the camera was handheld and constantly moving. Robustness to camera motion and orientation changes was confirmed by recalculating the homography matrix H for every frame based on the continuously tracked coloured markers. The system’s operational envelope was tested by varying the camera’s distance, as well as its pitch, roll, and yaw angles relative to Face B, to determine the limits of stable detection and accurate reconstruction. A mobile implementation, using *MATLAB Mobile* on a smartphone, was also performed to evaluate the system’s performance under hardware constraints.

## Results and discussions

### Light transmission and privacy behavior

**Fig. 5:**
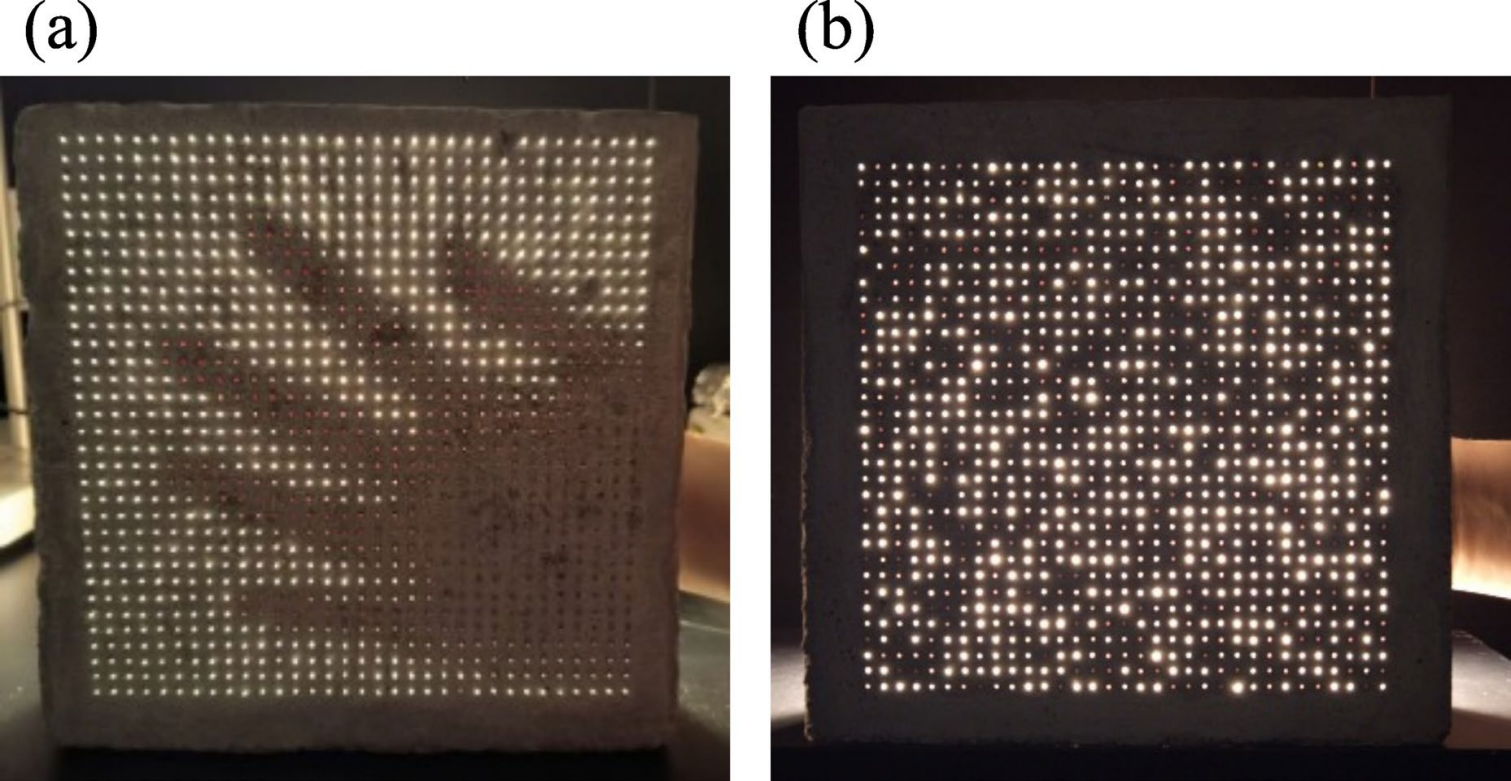
Optical response of semi-translucent mortar blocks: (**a**) parallel fiber configuration showing the visible shape of a hand behind the block, (**b**) random distribution configuration where the same hand becomes indiscernible.

Direct optical observation confirmed the functional difference between the two configurations (Fig. 5). The parallel arrangement resulted in coherent light channels that allowed the background object’s contour (a hand) to be clearly visible, a conventional outcome for parallel fiber composites. Conversely, the random distribution configuration produced a diffuse, unstructured light pattern, although the same object (a hand) was positioned behind the block. Under identical lighting conditions, the background object was rendered indiscernible, confirming that the random fiber arrangement effectively diffuses transmitted light, successfully achieving visual privacy while preserving light transmission. This behavior represents a topological transition from a highly anisotropic network (parallel fibers), characterized by a maximized orientation index along the transmission axis, to a stochastically isotopic network where the lack of structural connectivity correlations disrupts image coherence^35^. This functional divergence establishes a clear and demonstrable control over the transparency-privacy balance through fiber arrangement alone.

### Digital reconstruction of the fiber network

**Fig. 6:**
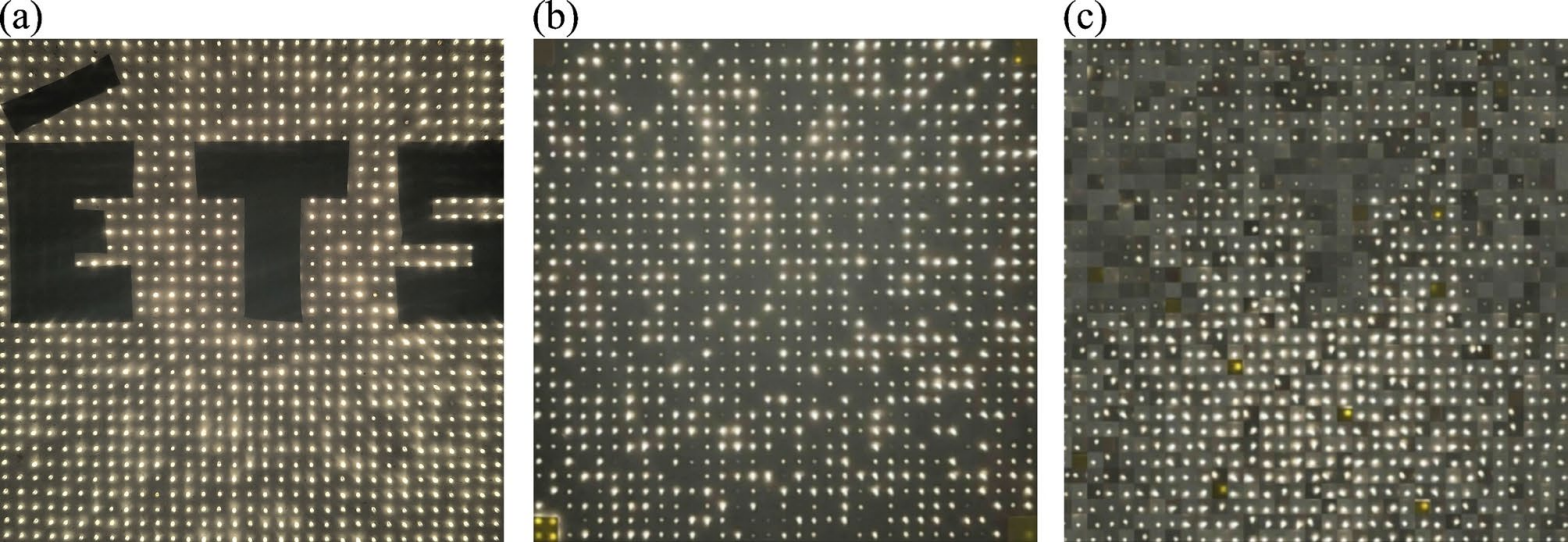
Digital reconstruction of the random fiber network revealing hidden letters “ÉTS”. (**a**) Background letters behind the block (face A), (**b**) view from the block surface (face B) before reconstruction, (**c**) Face B after digital reconstruction, where letters become clearly discernible.

Following the initial observations of light transmission, the optical fiber network in the random distribution specimen was digitally reconstructed based on the recorded input-output mapping. Each fiber’s exit point was identified and associated with its corresponding patch on the surface. These patches were then reassigned according to the virtual reconstruction, producing an ordered image that reveals objects hidden behind the block. As an example, the letters “ÉTS” placed behind the specimen became visible once the network was reconstructed (Fig. 6). The reconstruction confirmed that, although the physical block diffuses light and conceals shapes, the underlying fiber paths can be computationally realigned to transmit coherent visual information. This functionality can be restricted to authorized users by limiting access to the decoding scheme (i.e., the input-output mapping and related code), while maintaining privacy from external viewers. This establishes a “one-way privacy” functionality in the proposed translucent composite.

A quantitative analysis of the reconstructed image error (Fig. 6c) confirms the fidelity of the hidden image retrieval. The reconstruction error ($\:{E}_{rec}$) was calculated by quantifying the number of incorrectly classified fibers relative to the ground truth mask $\:{(S}_{mask})$, in Eq. (5).

5 $$\:{E}_{rec}=\frac{\sum\:\left|{S}_{rec}-{S}_{mask}\right|}{{N}_{total}}\cdot\:100$$

Where $\:{N}_{total}$ is the total number of fibers (1089). Specifically, in the “ÉTS” test, the ground truth established that 208 fibers should be classified as masqueraded. The digital reconstruction correctly identified 202 of these fibers as “off”, resulting in six misclassified fibers. This analysis yielded a minimal relative pattern error of approximately 2.88% within the dark-fiber subset. This classification error corresponds to a total system error of only 0.55% when normalized by the total fiber count.

This small deviation may result from minor light leakage between neighboring fibers or slight misalignments during the imaging process, or limitations in the digital reconstruction algorithm itself, such as discretization and rounding effects during patch repositioning. Similar to the boundary noise removal strategies employed in high-precision granular reconstruction^40^, future iterations of this algorithm could incorporate morphological filtering to further mitigate these discretization artifacts. The slightly uneven or irregular overall alignment observed in Fig. 6c results from the digital mapping scheme. During the automated mapping process, a quadrangular area was assigned to each individual fiber, which means that not all fibers remained perfectly centered within their assigned region. When these patches are digitally reconstructed, the spacing between neighboring fibers can appear locally wider or narrower. This minor positional distortion does not affect the visual information, as the letters “ÉTS” remain clearly recognizable after processing.

The reconstruction process also highlighted the spatial accuracy of the mapping. Patches (i.e. small image blocks corresponding to individual *n*×*n* “pixel-fibers”) were correctly repositioned according to the input-output dataset, confirming that the algorithm preserves the geometric consistency of the fiber network. These results illustrate that the random fiber distribution achieves the intended privacy effect physically, while the digital reconstruction provides a reliable method to selectively recover visual information behind the translucent composite.

**Fig. 7:**
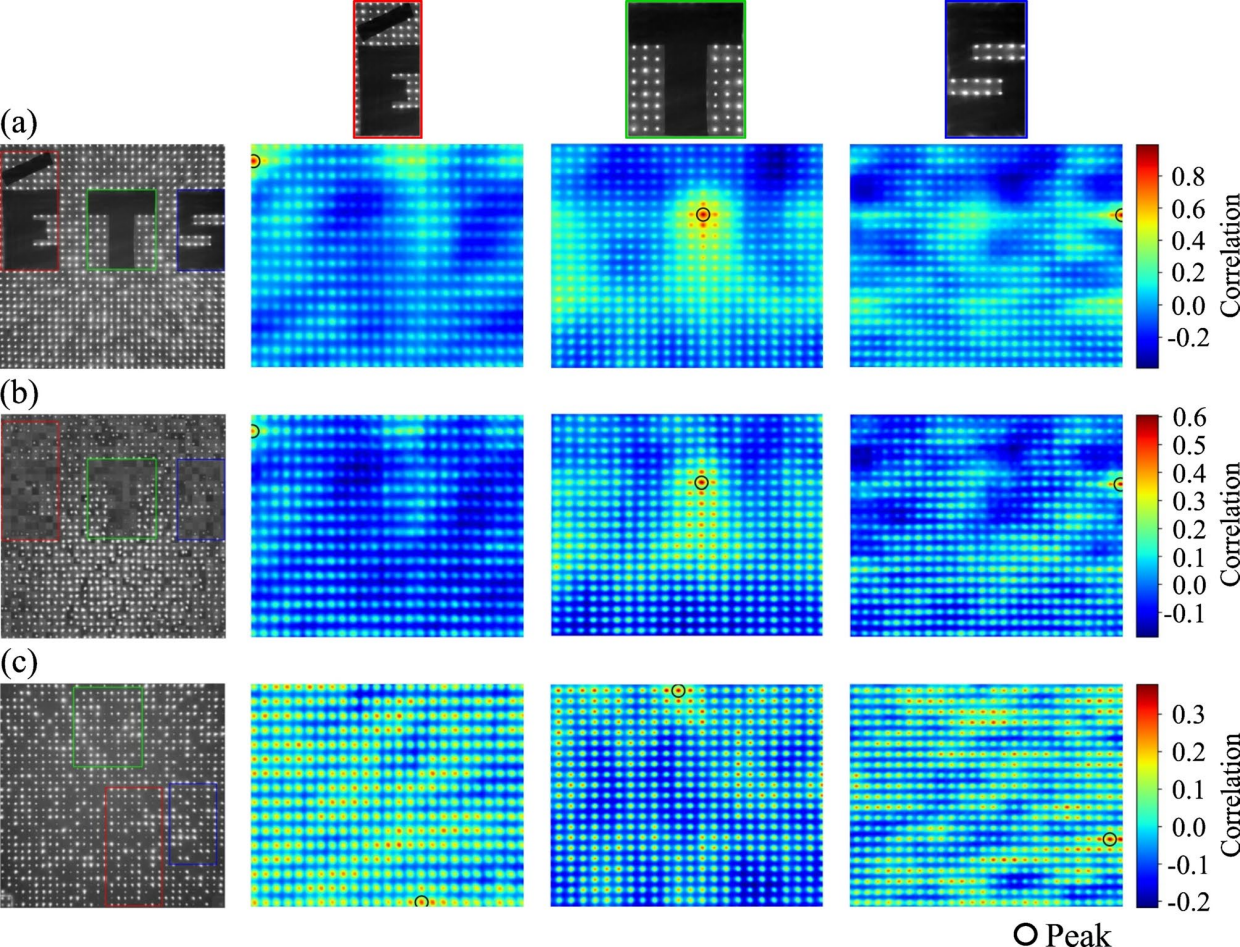
NCC maps for template matching across image configurations. The circles highlight the location of the peak correlation. (**a**) Reference image. (**b**) Reconstructed image. (**c**) Privacy-preserving image. The columns show the correlation maps for templates “E”, “T”, and “S”, respectively.

The fidelity of the reconstructed images and the effectiveness of the privacy-preserving configuration were assessed by analyzing template-image similarity using NCC. Templates corresponding to the letters “É”, “T”, and “S” were extracted from the reference image and correlated with the reference, reconstructed, and privacy-preserving images, generating correlation maps (Fig. 7). The maximum NCC value in each map was recorded as the primary measure of feature similarity.

Table 1 summarizes the maximum NCC results. As expected, the reference image shows the highest correlation values, confirming the clear presence of the features and establishing a robust baseline for comparison. In the reconstructed image, the templates were again successfully identified, yielding intermediate NCC scores. These values indicate that the reconstruction partially recovered the structural information corresponding to the templates. The letters are visually recognizable, but the reduced NCC scores reflect the loss of high-frequency fidelity due to minor reconstruction imperfections, such as patch misalignment or quantization effects (Table 2).

**Table 2:** Maximum NCC scores for template matching.

	E	T	S
Reference	0.890	0.992	0.994
Reconstructed	0.451	0.606	0.599
Privacy-preserving	0.298	0.380	0.338

Conversely, the privacy-preserving image shows the lowest correlation scores, demonstrating that the templates could not be reliably detected. As observed in the correlation maps, the few localized matches found correspond only to noise artifacts, failing to form recognizable shapes, thus quantitatively confirming the effectiveness of the privacy mechanism. Overall, the NCC analysis successfully separates the partially recovered information in the reconstructed image from the obfuscated and protected features in the privacy-preserving configuration.

**Fig. 8:**
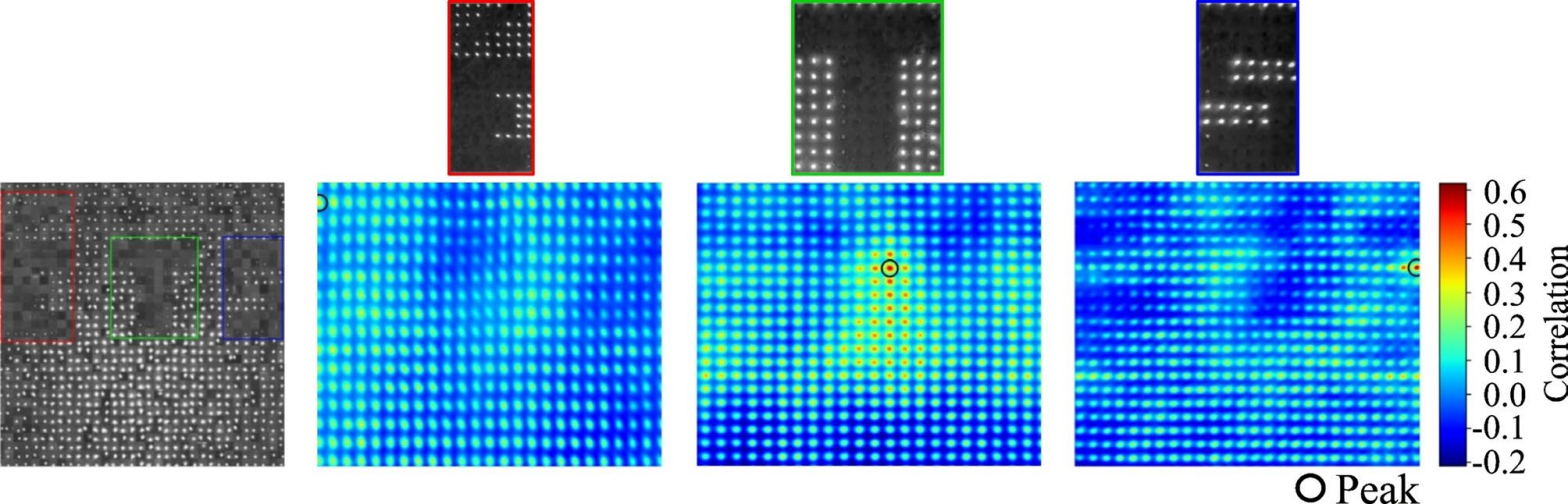
NCC analysis for template matching in parallel fibers template with reconstructed configurations.

For a quantitative comparison between configurations, we correlated the parallel fiber templates (“É”, “T”, “S”) against the reconstructed random-fiber image (Fig. 8). The results show that the correlation scores for “T” (0.594) and “S” (0.620) remain similar to those obtained from the reconstructed image (0.606 and 0.599, respectively). This moderate correlation suggests that the reconstruction process, even when targeting a random fiber configuration, retains sufficient generic structural information to allow successful template matching from the parallel configuration.

**Fig. 9:**
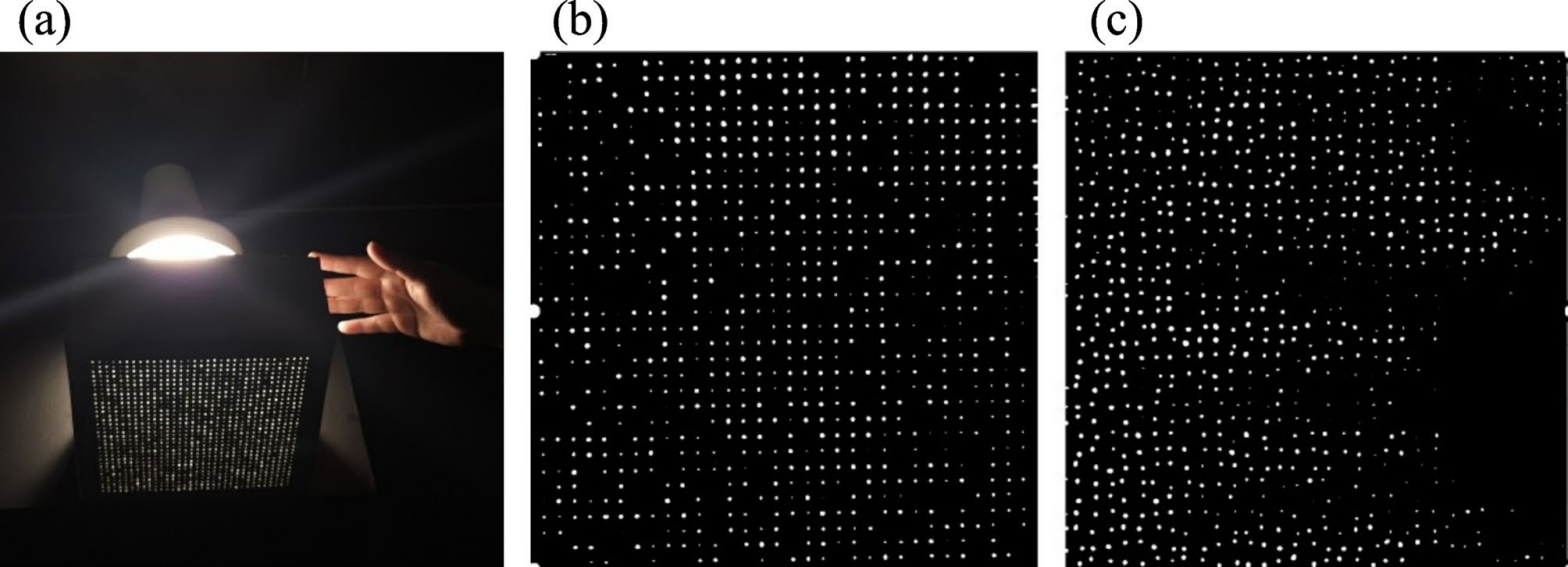
(**a**) Setup and specimen. Binary-converted frames selected from the digital reconstruction of hand movement behind the random-path specimen: (**b**) view without digital reconstruction, (**c**) digitally reconstructed frame.

To further assess dynamic performance, a hand was positioned behind the block (face A) and moved from a closed to an open posture in less than half a second. The motion was captured as a video sequence. After digital reconstruction, the opening and closing movements of the hand were clearly recognizable in the reconstructed view of face B (Fig. 9, static frames shown, full sequence available in Supplementary information). Although individual fibers may exhibit minor misassignments during rapid motion, the overall shape and movement of the hand remain clearly distinguishable, with the dynamic features most evident in the full video sequence. This test demonstrates that the reconstruction algorithm can reassign light patches under rapid motion, confirming the temporal fidelity and spatial accuracy of the method.

It is important to note that the mobile implementation using *MATLAB Mobile* achieved an average processing time of approximately 8 s per frame, indicating that the real-time performance is currently limited by the processing power of the handheld device. This latency does not reflect the efficiency of the core algorithm, which remains lightweight, requiring only homography calculation, image segmentation, and a simple pre-calibrated matrix lookup (M) for permutation. This approach differs from 3D reconstruction frameworks that rely on computationally intensive Large Vision Models^41^ or Optimal Transport^40^ algorithms to infer complex internal structures from 2D data. The proposed method employs a pre-calibrated lookup matrix tailored for the fixed optical network, enabling near real-time digital reconstruction of hidden visual information. The observed delay is mainly due to the interpreted research environment and non-optimized cloud communication rather than algorithmic limitations. The *MATLAB Mobile* platform was selected as an easy-to-use development and prototype testing platform for this proof-of-concept study, and allowed for the rapid algorithm prototyping and testing of the core digital-optical principle (the M vs. H correction).

For any practical, real-world application, this processing time can be drastically reduced by porting the reconstruction algorithm to compiled native code (e.g., C++ / OpenCV) and by leveraging on-device GPU acceleration. Overall, the results illustrate that the random fiber distribution achieves the intended privacy effect physically, while digital reconstruction provides a reliable method to selectively recover visual information behind the translucent composite.

### Augmented reality visualization and geometric robustness

Building upon the reconstruction process, an augmented-reality reinjection technique was implemented. The reconstructed image was inverse-transformed through the same homography used in calibration and superimposed on the live camera feed. This procedure enabled real-time visualization of the hidden object directly on the surface of the specimen. The reinjection remained stable for moderate camera movements, confirming that the homography maintained geometric consistency within the defined calibration domain. Minor misalignments were occasionally observed near the frame borders due to interpolation artifacts, but overall alignment was preserved. This experiment demonstrated that the random-fiber composite can operate as a physical-digital interface, where hidden information is accessible only through computational decoding and reintegration.

**Fig. 10:**
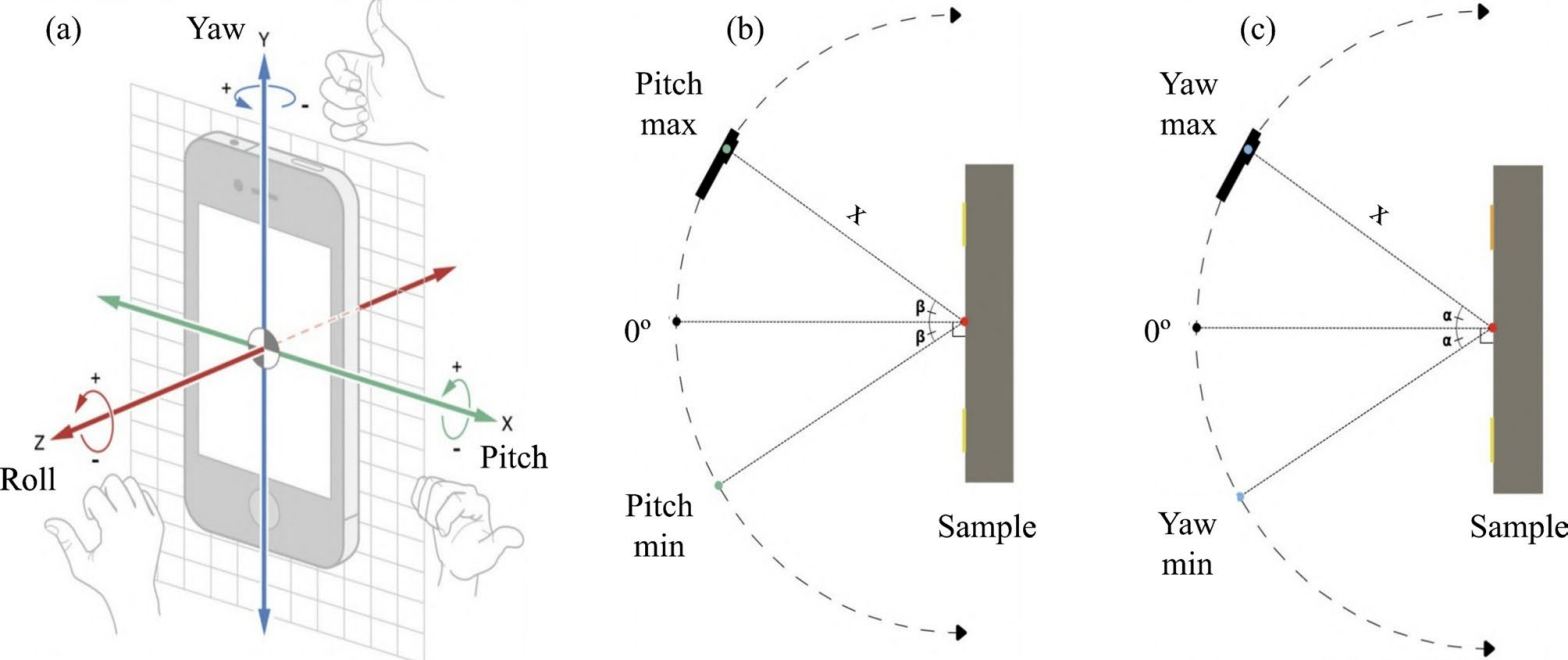
Camera orientation and calibration tests: (**a**) roll, pitch, and yaw relative to the smartphone axes, (**b**) side view of the yaw test, (**c**) top view of the pitch test.

To quantify the functional limits of the system, the spatial and angular dependencies of the calibration stability were analyzed. The system’s operational envelope was determined by monitoring the integrity of the marker detection and the accuracy of the homography transformation. First, the angular stability was quantified by testing the camera’s orientation across three axes: roll, pitch, and yaw (Fig. 10). The system successfully maintained accurate calibration for rotation angles up to approximately ± 35º in both pitch and yaw. Beyond these angular limits, the projection error of the markers increased progressively, producing small inaccuracies in the homography estimation and subsequent patch realignment.

**Fig. 11:**
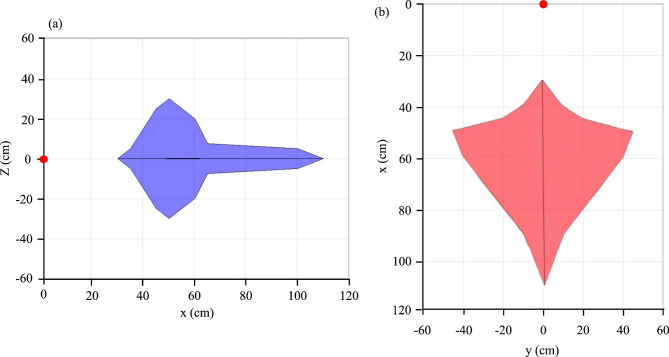
Spatial operational limits. Schematic representation of the stable viewing domain. The red circle marks the central axis of the translucent composite.

Subsequently, the viewing distance was tested by varying the camera’s position relative to face B, which revealed stable detection and accurate reconstruction for distances ranging from 30 to 110 cm (Fig. 11). Within this operational range, the mean geometric error remained below 3%, similar to Zeng et al.^54^, ensuring that reconstructed images preserved their correct proportions and spatial correspondence. These tests confirm that the system tolerates moderate camera motion and rotation, which is essential for practical, handheld acquisition. Further optimization of marker design and adaptive homography algorithms could extend this operational range to larger or more complex surfaces^53^.

## Conclusions

This study presented the development and experimental validation of a novel cementitious composite that integrates optical privacy and digital recoverability within a single material system. By embedding a traceable, randomly distributed network of optical fibers into an LC^3^-inspired mortar, the composite effectively diffused light, concealing shapes and achieving visual privacy while maintaining the potential for computational decoding. The core innovation consists of computationally reverse-engineering the light path. Using a pre-recorded input-output mapping matrix and homography-based calibration, a digital algorithm was able to reconstruct coherent images from the diffused light pattern. Validation tests demonstrated the system’s robustness in dynamic environments, with stable reconstruction achieved across an operational envelope defined by viewing distances of 30–110 cm and rotation angles up to ± 35º (pitch/yaw), maintaining a mean geometric error of less than 3%.

Despite the promising results, some limitations were identified that suggest directions for future work. The current study employed a 33 × 33 fiber grid (1089 fibers) in a lab-scale specimen, which is sufficient for demonstrating the concept but presents clear challenges for real-world applications. Scaling to larger panels with higher fiber densities introduces significant fabrication complexity and increases the computational load for digital reconstruction. In addition, larger or denser fiber networks may experience increased light attenuation and bending losses, although for typical lengths of a few meters, these effects remain relatively small, with attenuation on the order of 2 dB over 10 m for the fibers used in this study, which is not a limitation in the current application.

From the algorithmic perspective, the current implementation relies on a pre-calibrated lookup matrix and homography-based mapping, which is lightweight but still limited by the processing power of handheld devices. Real-time performance is therefore constrained, and further optimization is necessary to allow fast on-device computation. Minor deviations in patch alignment can also occur due to marker detection errors or environmental variations, which could affect reconstruction fidelity under more dynamic conditions.

To address these challenges and enable larger-scale applications, a modular approach is proposed, in which multiple privacy blocks with independently randomized fiber mappings can be assembled to form larger structures or walls. This strategy allows the benefits of the random fiber configuration to be scaled while maintaining manageable fabrication complexity, ensuring that each module preserves its optical privacy and traceable digital reconstruction. Future work should also explore the optimization of the computational pipeline through migration to compiled native code, adaptive marker detection for enhanced calibration robustness, and strategies to further reduce alignment errors or compensate for environmental influences. Additionally, studies on fiber layout optimization, alternative fiber types, and automated placement techniques could help scale the system while maintaining high fidelity and optical performance.

## Supplementary Information

Below is the link to the electronic supplementary material.

Supplementary Material 1

## Data Availability

The datasets generated and/or analysed during the current study are not publicly available due to intellectual property considerations but are available from the corresponding author upon reasonable request.
